# Nanotube Functionalization: Investigation, Methods and Demonstrated Applications

**DOI:** 10.3390/ma15155386

**Published:** 2022-08-05

**Authors:** Marianna V. Kharlamova, Maksim Paukov, Maria G. Burdanova

**Affiliations:** 1 Centre for Advanced Material Application (CEMEA), Slovak Academy of Sciences, Dubravska Cesta 5807/9, 845 11 Bratislava, Slovakia; 2 Center for Photonics and 2D Materials, Moscow Institute of Physics and Technology, Institutsky Lane 9, 141700 Dolgoprudny, Russia; 3 Institute of Solid State Physics, Russian Academy of Sciences, 142432 Chernogolovka, Russia

**Keywords:** carbon nanotubes, covalent modification, non-covalent modification, filling nanotubes, substitution of atoms

## Abstract

This review presents an update on nanotube functionalization, including an investigation of their methods and applications. The review starts with the discussion of microscopy and spectroscopy investigations of functionalized carbon nanotubes (CNTs). The results of transmission electron microscopy and scanning tunnelling microscopy, X-ray photoelectron spectroscopy, infrared spectroscopy, Raman spectroscopy and resistivity measurements are summarized. The update on the methods of the functionalization of CNTs, such as covalent and non-covalent modification or the substitution of carbon atoms, is presented. The demonstrated applications of functionalized CNTs in nanoelectronics, composites, electrochemical energy storage, electrode materials, sensors and biomedicine are discussed.

## 1. Introduction

The carbon nanotubes (CNTs) are modified by different routes, such as covalent and non-covalent modification of the outer surface, the substitution of atoms or the filling of CNT channels [1,2,3,4].

Covalent functionalization (adsorption) requires reactive species that can form covalent adducts with the sp^2^ carbon of CNT. The main advantage of the covalent functionalization of CNTs is that it is stronger than non-covalent interactions, however, it requires damage to the sidewalls. Unlike non-covalent functionalization, it changes the electronic structure, resulting in the irreversible loss of double bonds. These changes can affect conductivity properties and therefore, some applications [5,6,7,8,9,10].

Surface modifications of SWCNTs are not only achievable by covalent functionalization, but also by non-covalent methods. Known examples of molecules spontaneously assembling into micelles around SWCNTs are polymers [11], polycyclic aromatic compounds [12,13,14,15,16], biological species [17] and surfactants [18,19,20].

Covalent incorporation of foreign atoms within the CNT lattice is proposed for the effective engineering of CNT properties by consequent doping. Numerous materials have been used for SWCNTs’ substitution, such as N, B, P, Si, Cl or Ar. The important parameter in this case is the controlled concentration of the embedded atoms. Substitutional doping introduces strongly localized electronic features in the valence or conduction bands of CNTs, which makes it different from doping. Different methods can be utilized for the substitution of atoms: arc-discharge, laser-ablation, CVD, chemical reactions and plasma treatment [6].

This review is dedicated to the methods of nanotube functionalization. Section 2 discusses the microscopy and spectroscopy of functionalized CNTs. Section 3 demonstrates the update on covalent and non-covalent modification, the substitution of carbon atoms in CNTs and the filling of CNTs. Section 4 presents applications of functionalized CNTs.

## 2. Microscopy and Spectroscopy of Functionalized Carbon Nanotubes

There are several methods of the functionalization of carbon nanotubes:

(a) exterior covalently attached functional groups,
(b) exterior physisorbed moieties,
(c) heteroatoms directly incorporated into the sp^2^ mesh,
(d) endohedral filling of SWCNTs.

Each of these distinct techniques is a well-established research area with a distinct origin and history. To which extent the different techniques are applicable depends greatly on the intended modification and the requirements of a specific application. They were all tailored toward specific aims in accordance with their unique weaknesses and strengths. In this context there cannot be a meaningful classification according to general advantages and disadvantages. Yet, for a novel use case, the features of the existing techniques can be evaluated according to the current goals and requirements.

In this section we discuss the microscopy and spectroscopy of fluorinated CNTs. Attaching various functional groups (typically by covalent bonding) constitutes exterior chemical modification of the walls of SWCNTs [21]. The inception and further development of this kind of modification was and is strongly motivated by making SWCNTs soluble. This is a key requirement for virtually any large-scale bulk material processing. Pristine SWCNTs are completely insoluble in aqueous solutions or any other known organic solvents [21,22]. They can be dispersed temporally by ultrasonic agitation, but will quickly precipitate after the sonication is stopped [21]. Surface modification is also the way to let SWCNTs specifically interact with many different compounds [17,23,24,25,26,27,28,29,30,31,32,33,34,35,36,37,38,39,40,41,42,43]. Engineering nanotube-based supramolecular complexes opens up new avenues for the design of nanodevices. If suited polar groups are attached to the surface of SWCNTs, they can be dissolved in aqueous biological systems [21]. The correct choice of functional groups can also lead to changes in their electronic properties [22].

**Figure 1:**
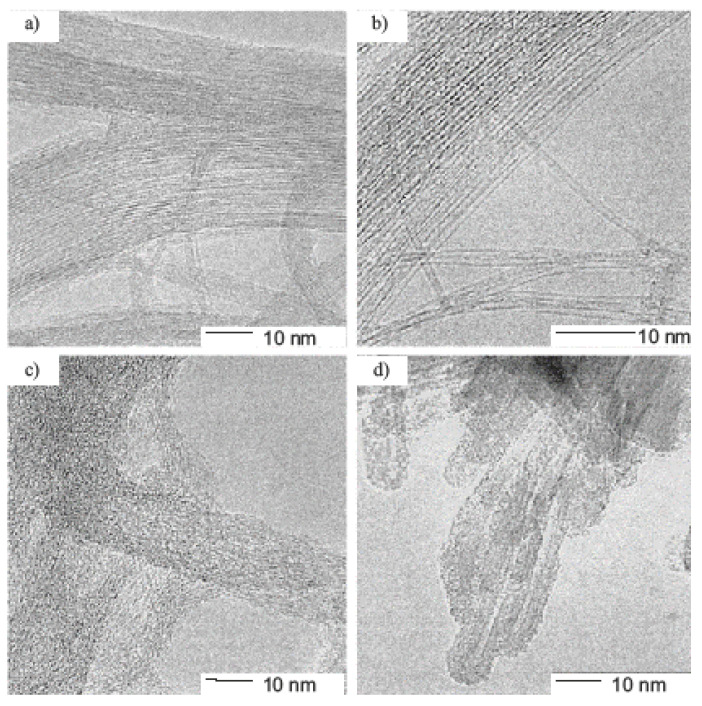
TEM micrograph of purified SWCNTs (**a**). TEM micrograph of SWCNTs after fluorination at 325 °C (**b**). TEM micrographs of SWCNTs after fluorination at 500 °C reveal a mixture of defective bundles (**c**) and MWCNTs (**d**). Scale bar, 10 nm. Reprinted with permission from Ref. [44]. Copyright 1998 Elsevier.

Figure 1 shows the transmission electron microscopy (TEM, JEOL model 2010 TEM using 100 keV beam energy, Tokyo, Japan) image of the purified SWCNT starting material (A), fluorinated SWCNTs at 325 °C (B) and fluorinated SWCNTs at 500 °C (C, D) [44]. Single-walled carbon nanotubes were produced by the dual-pulsed laser vaporization of Co/Ni doped graphite rods. The starting material was a very clean SWCNT. Fluorination at 325 °C leaves the SWCNTs largely intact. Fluorination at 500 °C is obviously very destructive. The structures in Figure 1C are morphologically still reminiscent of the bundles of the SWCNT, but they are clearly disordered internally. The structures in Figure 1D are also a common product of the high temperature fluorination. They appear to be hollow and are more reminiscent of defective MWCNTs [44].

**Figure 2:**
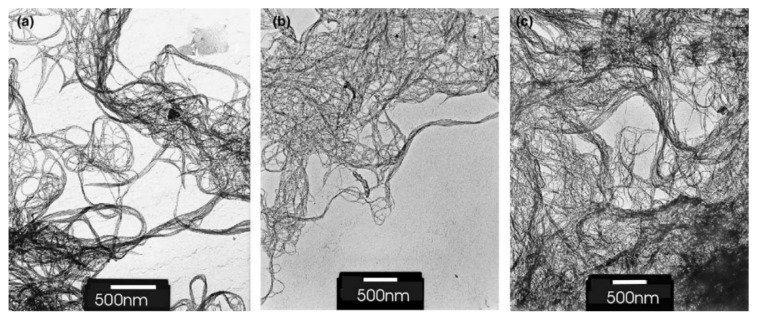
TEM images of samples at 100 kV: (**a**) control unexposed samples; (**b**) exposed to SF_6_ at −200 V bias; (**c**) exposed to CF_4_ at -200 V bias. Reprinted with permission from Ref. [45]. Copyright 2004 Elsevier.

In Ref. [45], SWCNTs were functionalized using a reactive ion etching system with CF_4_ and SF_6_ plasmas. Figure 2 shows the TEM images of the pristine SWCNTs and the samples exposed to SF_6_ at −200 V bias and CF_4_ at −200 V bias. The SWCNTs’ network was similarly functionalized in all of the shown samples, forming bundles due to van der Waals interactions. Meanwhile, the CNTs appeared to be unharmed.

**Figure 3:**
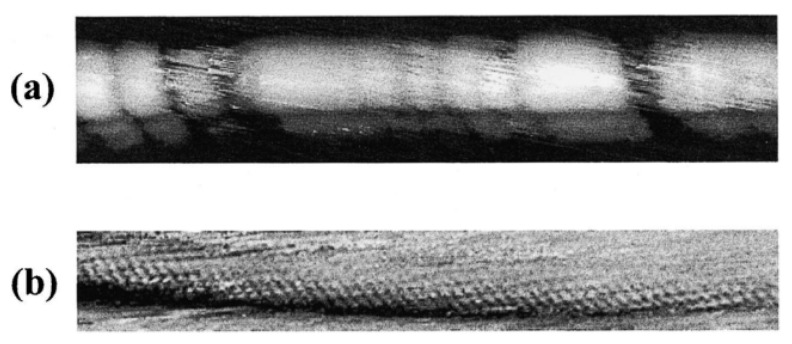
STM images of a SWCNT (**a**) after fluorination at 250 °C for 12 h. The bright regions correspond to sections on the tube covered by fluorine atoms. (**b**) A pristine single-walled carbon nanotube was deposited on an Au (111) surface grown on mica substrate and imaged with a C60-functionalized STM tip. The image is high-pass filtered Reprinted with permission from Ref. [46]. Copyright 2002 Elsevier.

**Figure 4:**
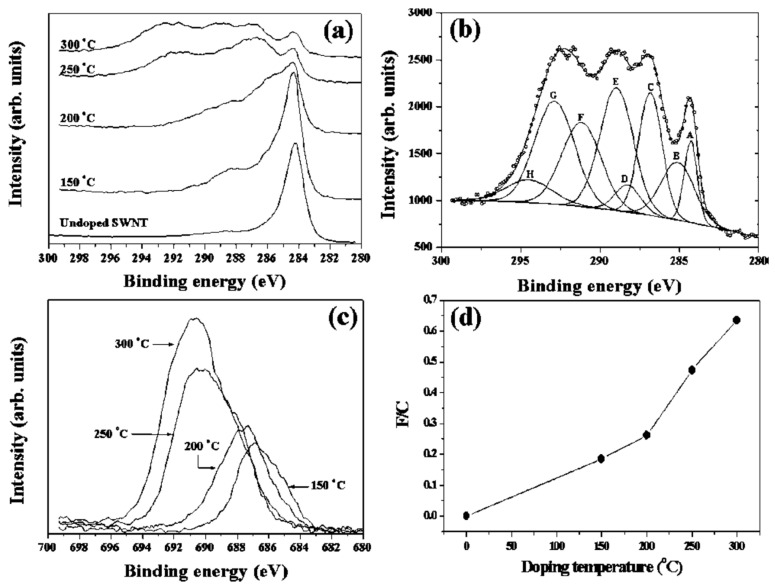
(**a**) C 1s XPS spectra of pristine SWCNTs and SWCNTs fluorinated at 150, 200, 250 and 300 °C. (**b**) Gaussian peak fitting of the C 1s XPS spectra of the SWCNTs after fluorination at 300 °C. The individual peaks are due to sp^2^ (A), sp^3^ (B), oxygen bonds (C–E) and fluorine bonds (F–H). (**c**) F 1s XPS spectra of the same fluorinated SWCNTs. (**d**) Atomic F/C ratio versus fluorination temperature Reprinted with permission from Ref. [47]. Copyright 2002 Elsevier.

Figure 3 compares scanning tunnelling microscopy (STM) images of a fully fluorinated (a) and a pristine SWCNT (b) [46]. The fluorinated SWCNT features sharp segmentation into irregular, short, dark sections separating longer, bright, axial sections. The bright sections are due to the piecewise fluorination. Atomic resolution is readily achievable and commonly reported for a wide range of scanning parameters, also covering those that were used for the pristine SWCNT in Figure 4b. Atomic resolution is not readily achievable for fully fluorinated SWCNTs, albeit the chiral twist is resolved [46].

CNTs were fluorinated at different temperatures ranging from 150 to 300 °C. The C 1s X-ray photoelectron spectra (XPS) of the pristine SWCNT in Figure 4a show the narrow asymmetric peak of the sp^2^ carbon at 284.3 eV [47]. The tail above 285 eV indicates a minor presence of sp^3^ carbon. Upon fluorination at increasing reaction temperatures, the sp^2^ peak becomes less intense as new peaks at higher binding energies arise and grow in intensity. The C 1s spectral range undergoes well visible changes. Each fluorination temperature has a clearly distinct fingerprint due to different chemical shifts. Figure 4b shows the line shape analysis of the C 1s spectrum after fluorination at 300 °C. The individual Gaussian peaks are identified as sp^2^ (A), sp^3^ (B), various oxygenation states (C–E) and also multiple fluorination states (F–H). The peaks, F, G and H at 291.2 eV, 292.9 eV and 294.6 eV, are ascribed to C-F, C-F_2_ and C-F_3_ bonds, respectively. The F 1s XPS spectra in Figure 4c shift to higher binding energies and grow in intensity with higher fluorination temperatures [47]. The F 1s peak position shifts abruptly above 200 °C from 687 eV to 691 eV. This marks the transition from semi-ionic fluorine to fully covalent C-F bonding. This interpretation is corroborated by the known F 1s position in covalent hexafluorobenzene at 691 eV. The C to F stoichiometry, as evaluated from integrated peak areas and the photoelectron cross section, increases monotonically and reaches about 0.65 at 300 °C [47].

**Figure 5:**
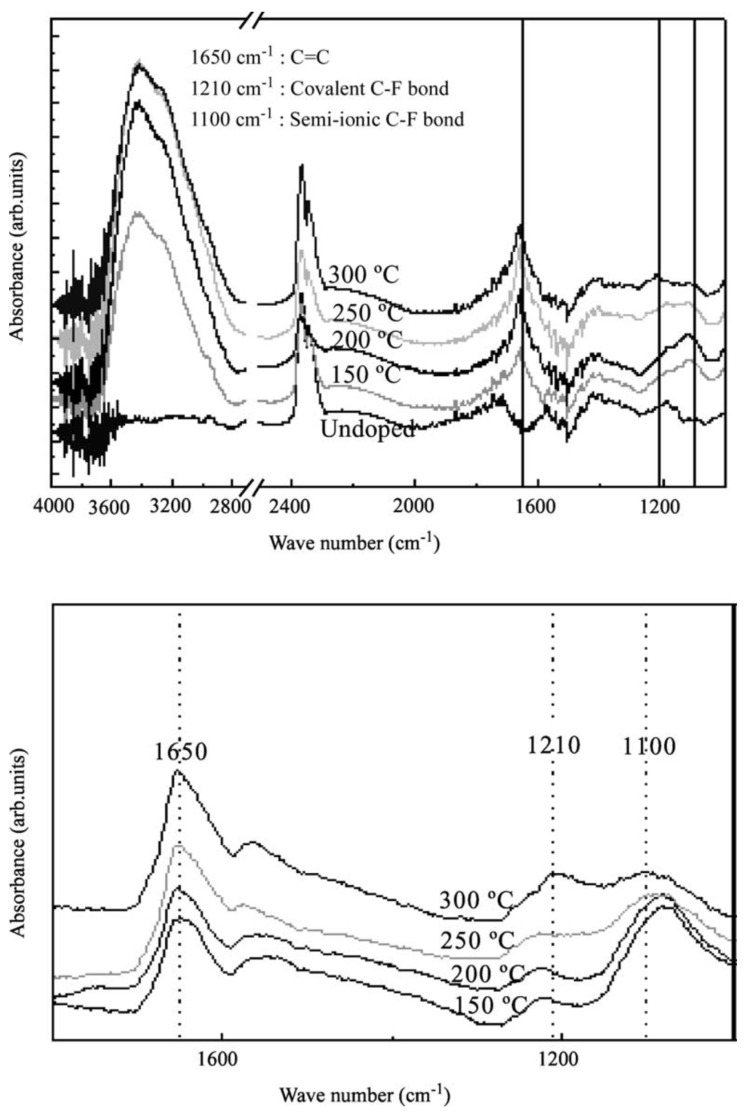
FTIR spectra of pristine SWCNTs and SWCNTs fluorinated at 150, 200, 250 and 300 °C. Reprinted with permission from Ref. [48]. Copyright 2003 Elsevier.

The Fourier-transformed infrared (FTIR) spectra in Figure 5 belong to pristine SWCNTs and different temperatures of fluorinated SWCNTs. The fluorination temperatures were 150, 200, 250 and 300 °C. The close-up in the lower panel shows only the spectra of fluorinated SWCNTs and reveals a decrease in the peak at 1100 cm^−1^ and an increase in the peak at 1230 cm^−1^ with increasing fluorination temperature. The first shrinking peak signifies the loss of ionic fluorine while the second growing peak confirms a gain of covalent C-F bonds. The broad bands around 2350 cm^−1^ and 3400 cm^−1^ belong to CO_2_ and OH groups.

**Figure 6:**
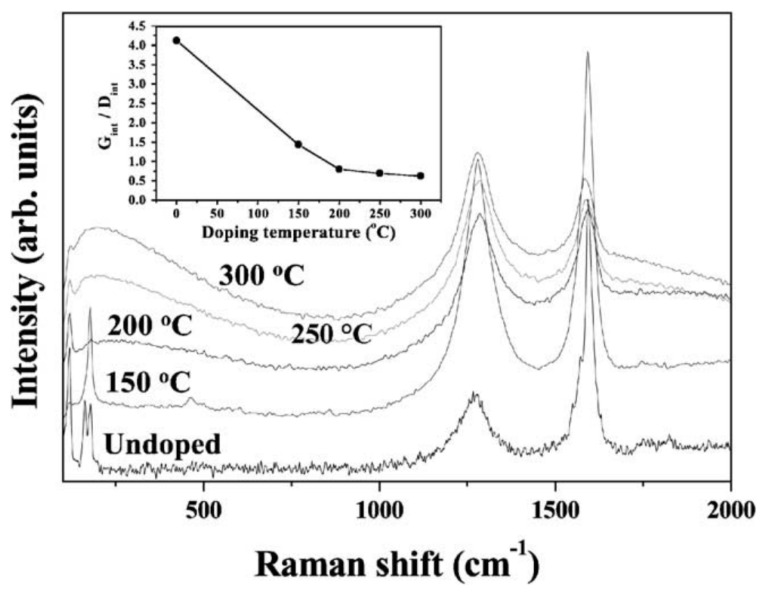
The Raman spectra of the pristine SWCNTs and the nanotubes fluorinated at 150, 200, 250 and 300 °C. The inset shows the dependence of the intensities of G and D modes on the doping temperature. Reprinted with permission from Ref. [48]. Copyright 2003 Elsevier.

Figure 6 presents the Raman spectra of undoped SWCNTs and fluorinated SWCNTs taken at various temperatures [48]. The undoped and fluorinated SWCNTs have a smaller peak near 200 cm^−1^, which corresponds to the SWCNTs’ distinctive breathing mode. It was shown that the breathing mode is suppressed due to doping. Broadband spectra consist of distinctive 1580 cm^−1^ and 1360 cm^−1^ (G and D band, respectively) peaks. As the fluorination temperature rises, the intensity ratios (I_G_/I_D_) decrease. This demonstrates the alteration in the bulk structural characteristics of CNTs after fluorination [48].

**Figure 7:**
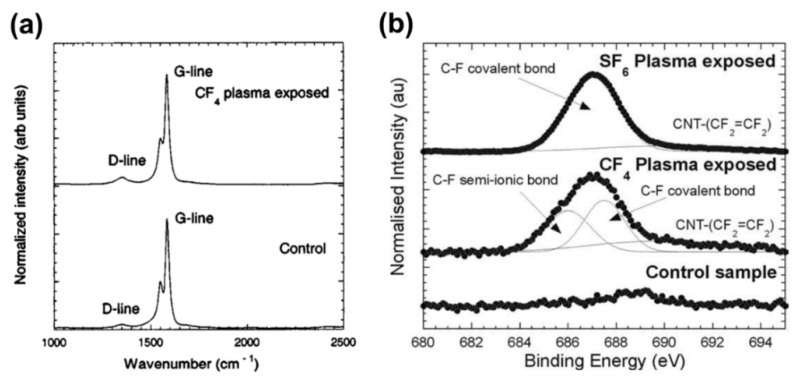
(**a**) Raman spectra of control and CF_4_ plasma exposed single-walled CNTs. (**b**) The deconvoluted F 1s peak for the SF_6_ and CF_4_ plasma exposed SWCNTs at −200 V for 1 min in comparison to the unexposed control sample. Reprinted with permission from Ref. [49]. Copyright 2004 Elsevier.

In Ref. [49], the impact of CF_4_ gaseous plasma treatment on SWCNTs was investigated. In particular, Raman spectroscopy showed that CNTs had more disordered sp^3^ bonds, associated with functionalization, as gas flow rates in the plasma and exposure duration in the plasma were increased (Figure 7a). XPS has been used as a means of detecting chemical species attached to the CNTs. The F 1s region of the XPS spectra of SWCNTs exposed to the CF_4_ plasma, the SF_6_ plasma and the pristine control sample are shown in Figure 7b. The tubes treated with CF_4_ plasma was fitted with two large Gaussian peaks, one at 686 eV in the semi-ionic region and another at 687.5 eV in the region which corresponds to covalent C–F bonding. Over all exposure conditions, the SWCNTs treated in SF_6_ plasma only needed one peak at 687 eV, toward the covalent area of the F 1s spectra. The weak sputtering effects cause the broad low intensity peak at 689.5 eV that is visible after both functionalization procedures.

## 3. Modification of Carbon Nanotubes

### 3.1. Covalent Modification

Defect functionalization is based on the attachment of the desired functional group to the existing or intentionally created defects on the CNT. In reality, CNTs are not ideal nanostructures, but rather contain 1–3% of defects that naturally occur during synthesis. Another type of defect can invariably be introduced during the preparation and purification stages. Ultrasonic treatment and shear mixing, widely used to disperse CNTs in solution, can introduce these defects. The effect of sonication is based on the cavitation effect that is able to break the CNT aggregates (bundles) in addition to the shear force effect. This causes the formation of the structural defects and eventually, their breakage. After defect introduction, the sidewalls are expected to be chemically active, thus improving the reactivity of the CNTs.

Oxygenated sites can also be considered defects. Chemical oxidation of nanotubes is mainly achieved by using either wet chemistry or gaseous oxidants. One way of creating defects and functional groups is oxidative damage to the nanotube framework via strong acids or oxidants, such as HNO_3_, H_2_SO_4_ or KMnO_4_, which leave holes functionalized with oxygenated functional groups as well as open CNT caps [50,51,52]. The oxidation can also be archived by using hydrochloric acid [53,54], hydrogen peroxide [7], potassium permanganate [55], oxidative persulfates [56] and hypochlorites [57]. In Ref. [52], it was shown that the oxidation of CNTs with concentrated HNO_3_ results in defect formation in addition to the opening of the ends of the nanotubes. Besides that, it stimulates the formation of the aromatic polycyclic compounds (i.e., fulvic acids on the surface), which can be removed by an alkali and then reversibly reabsorbed. This consequently results in the improvement of the dispersion of CNTs in the aqueous solution.

The gas phase functionalization of CNTs, including oxidation, was carried out. Here, carbon dioxide [58,59], water vapor [60], air, oxygen [61,62,63] and ozone [64,65,66] were used as oxidants. Water vapor is effective as an oxidizing reagent only when it is introduced into the reaction during CVD synthesis of CNTs [60]. It was shown that in this method, nanotubes are produced with a sufficiently large number of defects and oxygen content up to 42 wt.% in different functional groups. So far, the oxidation with oxygen in the air is the most effective method of oxidation as it proceeds with the simultaneous CNT cleaning, shorting and opening. In general, oxidation treatment leads to the p-type doping of CNTs, shifting the Fermi level towards the valence band.

Moreover, some functionalization methods are combined together. For example, in [67], the effectiveness of the same carboxylation of CNTs in a mixture of nitrogen-nitric acid (3:1) is accompanied by cavitation caused by ultrasonic action at room temperature. In addition, the effectiveness of hydrogen peroxide for the oxidation of CNTs increases with UV irradiation [68,69]. Air can be used for additional oxidization of CNTs pre-treated with acids [70].

It was also shown that the oxidation processes strongly depend on the curvature of the CNT’s wall and consequently, the diameter dependence [71,72,73]. Upon oxidative functionalization of CNTs, the affinity of their surfaces to various solvents and polymer matrices increases. The defects on CNTs created by oxidants are stabilized by bonding with carboxylic acid (–COOH) or hydroxyl (–OH) groups and ketone, alcohol and ester group formation. These groups can be used as precursors for further functionalization: silanization [74,75,76], esterification [77,78,79], thiolation, alkylation, arylation and biomolecule and polymer attachment.

CNT silanization is the interaction of oxidized CNTs with organosilanes. The silanization after oxidation usually takes place in a few steps. Firstly, the hydrolysis of silanes into silanols is performed. The creation of hydrogen bonds between oligomers and the surface hydroxyl groups of carbon nanofillers as well as the condensation of silanols into oligomers occurs in the next phase. Finally, Si-O-C bonds are created through the interaction of Si-OH groups with OH groups on CNTs. The 3-aminopropyltriethoxysi-lane has been widely used as a silane agent [80,81,82,83,84]. Lin et al. used comparable techniques to attach both polyethylene glycol and iron oxide in silica nanoparticles to the surface of CNTs [85].

Access to numerous CNT surface functionalizations is made possible by adding carboxylic and hydroxyl groups to CNTs. CNTs can be functionalized via esterification or amidation. Typically, carboxylic groups are activated to acyl chlorides by thionyl chloride, and then amidation and esterification reactions convert them to carboxamides (or carboxyesters) to introduce a variety of functional groups as nucleophiles, such as aliphatic or aryl amines, amino acid derivatives, ensims, peptides, or amino-group-substituted dendrimers [64,78,79]. Thiolation with sodium sulfite and sodium hydroxide and carboxylation with thionyl chloride are used to attach a similar thiol group [86]. The presence of carboxyl groups themselves leads to the reduction of van der Waals interactions between CNTs, which results in effective debundeling and separation into individual but shortened nanotubes.

The chemical manipulation of carbon nanotube sidewalls via fluorination and then defluorination is well developed [48,49,87]. It can be carried out efficiently in the presence of F_2_ at temperatures between 150 and 400 °C [88]. The interest is that the fluorine atoms can then be substituted by alkyl groups or by amines. Fluorinated CNTs are widely used for a wide range of chemical functionalization. For example, sidewall alkylation and arynation of fluorinated SWCNTs was performed using alkyllitium species, including methyl, ethyl, butyl, hexyl, octyl and dodecyl species. Amino groups can also be attached to the sidewalls of fluorinated CNTs [89]. These materials exhibit an excellent reinforcement effect in epoxy-based systems, which results in improved properties like mechanical robustness. The chlorination and bromination are another two methods that have been used for SWCNT functionalization. Chlorine applied to the functionalization of CNTs can be liquid precursors such as thionyl chloride, chloroform, trichloromethane, tetrachloroethylene or hydrochloric acid or solid precursors such as dichlorocarbene or chlorine gas precursors [90,91,92,93]. Purified CNT samples attached 1.8 at.% of bromine after two days in saturated bromine vapors at room temperature [94,95].

The most common approach to hydrogenation is wet chemistry which includes the dispersion of a mixture of lithium and CNT in diaminoethane [96]. The protonation of reduced lithium also reduces CNT with methanol, leading to the formation of hydrogenated SWCNTs [97]. As a result, the covalently bonded CH derivatives are formed. High-boiling polyomides were also used as the hydrogenation reagent for CNTs [98]. At room temperature, it has been demonstrated that hydrogenated SWNTs with covalent C-H bonds constitute a stable hydrogen storage vehicle [99,100,101].

CNTs functionalized with fluorescein or a fluorescent bioactive peptide can also effectively cross the cell membrane without significant damage of the membrane [102,103]. For therapeutic biomolecules, such as proteins, peptides, deoxyribonucleic acid (DNA) and siRNA, covalent functionalization has been widely reported [104]. For example, CNTs were used to deliver streptavidin into the cell [105]. It has been demonstrated that SWNTs may be covalently functionalized to peptide nucleic acid (PNA), an analog of uncharged DNA, and then hybridized to molecular wires containing complementary DNA [106]. There have been several reports of bovine serum albumin protein molecules adhering to f-MWCNTs in an aqueous buffer solution [107]. CNTs can introduce a DNA molecule into the cell nucleus which opens an opportunity to use CNTs in gen-delivery [108]. CNT, after covalent functionalization, can be used to carry peptide antigens [109]. Recently, the translocation of the RNA into breast cancer cells using non-functionalized SWCNTs has also been reported [110].

In general, the covalently functionalized CNTs are soluble in many organic solvents due to the attachment of polar or nonpolar groups. The main difficulty in developing covalent functionalization arises from sp^2^ to sp^3^ hybridization. This consequently leads to changes in mechanical, optical and electrical properties. On the other hand, the covalently functionalized CNTs open the road to many applications, which require the change in optical and electrical properties [111,112].

### 3.2. Non-Covalent Modification

The first commonly used non-covalent functionalization is surfactant based. These types of molecules result in the dispersion of the CNTs via physical adsorption. Both ionic and non-ionic surfactants have been used. The known surface charge of CNTs can be used to predict the colloidal stability of CNT solutions and the interaction (adsorption) mechanism with ionic surfactants. A larger negative surface charge and steric repulsion, for instance, are produced when CNTs and SDS interact through hydrophobic components, which increases the stability of the dispersion. Therefore, the purification procedure and tube wall functionalization, which influences its surface charge, may be used to regulate the adsorption mechanism of ionic surfactants [113]. While the polar portion of surfactants interacts with solvent molecules, the hydrophobic (nonpolar) portion is often oriented toward the surface of CNTs. The most popular surfactant for the dispersion of CNTs in aqueous media are sodium dodecyl sulphate (SDS), sodium dodecyl benzene sulfonate (SDBS), cetyltrimethyl ammonium bromide, Tween, Triton X and a siloxane polyether copolymer [114,115,116,117,118,119,120]. One of the important parameters for the dispersion of CNTs is CNT to surfactant concentration. For example, a homogeneous dispersion of CNTs was found at the optimum composition of 0.5 wt% CNT and 2 wt% SDS relative to water. Ref. [121] found that the surfactant dispersing power follows the order of SDS < Tween 20 < Tween 80 < TX-100. Meanwhile, it was shown that SDBS exhibited superior capability compared to TX100 and SDS in terms of its capacity to disperse SWCNTs [20]. However, it was reported that the dispersion of MWCNTs in three surfactants followed the order of SDS < CTAB < TX100 [122]. While surfactants are often necessary to utilize individualized SWCNTs in the dispersion, improved surfactant-removing protocols are needed to recover the initial SWCNT properties to ensure optimal performance. The washing with organic solvents [123,124], acidic oxidation [123] and annealing in inert [123] or oxygen atmospheres are used for this purpose [125].

Through π-π interfaces, aromatic compounds like anthracene, phenanthrene, pentacene, porphyrin, pyrene and fullerene interact with one another. Under mild sonication, the strong bonding of functional molecules and large systems allows for debundling and produces hydrophilicity. A CNT@Pyrene hybrid was developed, aimed at improvement heat transport due to the anion addition [126]. The Biotin−streptavidin recognition method is described in [127]. This complex showed stability at 18 days and an excellent loading capacity (about 1 streptavidin tetramer per 20 nm of SWNT). The anthracene groups were covalently added to the hydroxylated CNTs to aid in the orientation of the laccase active sites for direct electron transfer between enzymes and electrodes [128]. The excellent electrochemical catalytic property and stability of the SWCNTs functionalized with ferrocene was reported by Yang et al. [129]. Huang et al. have used ferrocene for the non-covalent functionalization of SWCNTs for L-glutamate detection [130].

The interaction of polymers with the CNT surface via wrapping was used to improve the dispersion of CNT in water and organic solvent and to further enable the separation of CNTs by metallicity and chirality and the separation of CNTs from carbonaceous and metal impurities [131,132,133]. The polyvinyl pyrrolidone, polyvinylidene fluoride, polystyrene sulfonate, cellulose derivatives, polypyrroles, glycolipids and redox polymers was successfully used for CNT dispersion [134].

Dispersions of MWCNT with the pluronic surfactant F-127 were made with the intention of examining the interactions of CNT with biological tissues [135]. A water soluble globular protein called bovine serum albumin adheres to the surface of CNTs and improves dispersibility [136]. Similar results were shown for MWNT−PEG composites [94]. It was concluded that functionalizing CNT with PEG enhanced the dispersion of the CNTs and increased their heat capacities [137,138,139]. To act as a shRNA delivery vehicle, a modified branched polyethylenimine was grafted to carboxylated single-walled carbon nanotubes (SWCNT) using a polyethylene glycol (PEG) linker [140]. CNTs functionalized with DNA were shown to have enhanced stability when used in different applications [141,142,143,144,145]. Similar to proteins, DNA form tight helices around CNTs, or non-covalent conjugates. DNA-functionalized CNTs were more effective than plain MWCNTs against malignant tissues [145]. Despite the popularity of research on SWCNT-DNA conjugates, only a small number of works have studied the removal of adsorbed DNA on SWCNTs using biological, physical and chemical treatments [103]. Numerous biological and pharmacological uses of chitosan, including drug delivery, cancer treatment and biosensors, have been researched [146,147,148]. Chitosan could effectively disperse the SWCNTs and provide a suitable biological interface for the immobilization of biomolecules [149]. The functionalized CNTs complexed siRNA and demonstrated 10–30% silencing activity and 10–60% cytotoxicity [150]. siRNA delivery using CNTs were successfully utilized [151].

Finally, we would like to discuss the mechanical interlocking of SWCNT as a possible route for non-covalent functionalization. In particular, in [152], the macrocycle precursors that were terminated with bisalkenes and wrapped around the NT by ring-closing metathesis were furnished with two-extended tetrathiafulvalene SWNT recognition units.

### 3.3. Substitution of Atoms in CNTs

Nitrogen has one more electron than C, which it can easily substitute for C atoms. There are two types of C–N bonds that occur: pyridine-type in which each N atom is bonded to two carbon atoms and a type in which the N atom is bonded to three carbon atoms. The first type results in either a p- or n-type doping depending on the concentration of N atoms; meanwhile, the second type leads to n-type conducting behavior. In situ doping techniques include high temperature arc-discharge [153,154], chemical vapor deposition (CVD) [155,156,157,158], chemically solvothermal procedures [159] and laser ablation methods [160,161]. Also, n-type doping can be archived by various post-treatment methods [162,163]. The post-thermal treating of oxidized CNTs with ammonia was performed [164]. Doping of isolated SWNTs with nitrogen was achieved by mixing appropriate amounts of acetonitrile with xylene. The 2–6 at.% of nitrogen in the SWNTs was observed in a controllable manner [165].

The halogens, such as fluorine, chloride and bromide, can also be attached to the sidewall of CNTs. As mentioned in Section 1, fluorination can effectively be used for further covalent functionalization. The fluorination of CNTs, the formation of so-called fluorotubes, is usually performed by temperature treatment in a molecular fluorine environment at 150–600 °C. The precise temperature control results in the control of the activity of the substitution, and therefore, in the degree of the fluorination. The heavily doped CNTs were produced and showed increased solubility in alcohols [166]. The comparison between graphene and CNT fluorination suggested that the curvature resulted in a weakening of the C−F bonding covalence [167]. By diluting fluorine with nitrogen at a ratio of 4:1 and treating SWCNTs with such a mixture at 250 °C for 10 h, the tubes were fluorinized with the stoichiometry of CF_0.43_. The heating resulted NTs under He with temperatures ranging from 100 to 400 °C desorbed the fluorine [168]. Hydrazine defunctionalizes fluorinated nanotubes, allowing for the recovery of intact CNTs after treatment. Interestingly, fluorinated single-walled nanotubes were highly insulating. The fluorination has been studied using various synthesis parameters [169,170].

The commonly used alternative method for CNT functionalization via substitution is chlorination. Usually, the source of chlorine comes from liquid precursors such as thionyl chloride, chloroform, trichloromethane, tetrachloroethylene or hydrochloric acid or solids such as dichlorocarbene or gaseous precursors such as Cl_2_. The removal of metal catalysts and amorphous carbon residues from CNT has been reported to be accomplished using Cl_2_ gas and Cl_2_ aqueous solution treatments. Simultaneously, this protocol introduces chlorine but with a relatively low amount [171]. The chlorine substitution with the concentration of 2.5 atom % on SWCNTs was shown by using electrochemical functionalization in a HCl and KCl aqueous solution [172]. A similar concentration (up to 2.9 atom %) of chlorine was introduced through treatment with SOCl_2_ under a high temperature and pressure with prolonged reaction times of up to 14 days [173]. Recently, carbon tetrachloride cold plasma was shown to produce chlorinated CNTs [174]. Chlorine is fixed to the nanotube’s edges and flaws as a result of the operation.

A method of purification for CNT polluted by carbon impurities was used because of the low sensitivity of CNTs to bromination. However, the C−Br bond is more reactive than the comparable iodinated fluoro- or chloro-derivatives. The bromination of CNTs required relatively difficult to achieve conditions as compared to other carbon nanostructures. With bromine in the liquid, gas or plasma phases, it can only be done by electrochemical, electrothermal or microwave processes at a higher temperature. Despite the improvement that has been shown, bromination causes a small amount of CNT degradation and fragmentation [175]. Bromine-containing SWCNTs were demonstrated to have metallic properties [176]. In [177], the CNTs were combined with liquid bromine for 10 days at 55 °C and then repeatedly washed with CCl_4_, carbon tetrachloride. The perpendicular arrangement of bromine atoms in relation to the CNT surface was readily seen using TEM. Br atom conjugation was made possible in the graphite structure of CNTs (2.8 % weight) by electrochemical oxidation of inorganic salts such as KBr [178]. Additionally, CNTs that had already been treated in aqueous solution were brominated using microwaves. A high Br atom concentration of 5–8 percent by weight was archived [179]. Elemental bromine and a group of Lewis acids (BBr3, BF3-Et2O, AlBr3, FeBr3, ZnBr2, etc.) may also be used to chemically brominate CNT, producing materials with 10–20% wt Br content. According to Bulusheva, the suction of CNT in liquid Br yields the C_17_Br stoichiometry. The covalent bromination process with a total weight of 4.9% in CCl_4_ solution at microwave irradiation and a cool CH_3_Br plasma were described in [180]. N-bromosuccinimide, NH_4_NO_3_/NBS and Br_2_ were used in an electrophilic addition and radical reaction with UV light or conventional heating to archive the effective bromination [181]. In comparison to other methods, this one was proven to be the most mild, delicate, ecologically friendly and quick. The effective bromine doping of single-walled carbon nanotube films under ultrasound was recently published [182].

The literature has covered a lot of various substitution methods, including the substitution by I, S, Si, Se, P-N and Ar. Another technique for changing the graphitic structure of CNTs is iodination. First, a modified Hunsdiecker reaction was used to carry it out [183]. It was found that Cl, Br and I easily produced halogenated CNTs at high temperatures and pressures. The CNTs have also been doped with iodine atoms using the straightforward solvent thermal reaction process [24] and their characteristics have been examined [184,185]. In order to illustrate the replacement of impurities in both materials, mono- and divacancies were produced in graphene and SWCNTs using the Ar plasma treatment [186]. Using STEM and other techniques, the Ar ions were plainly visible on the surface of CNTs.

## 4. Applications

**Figure 8:**
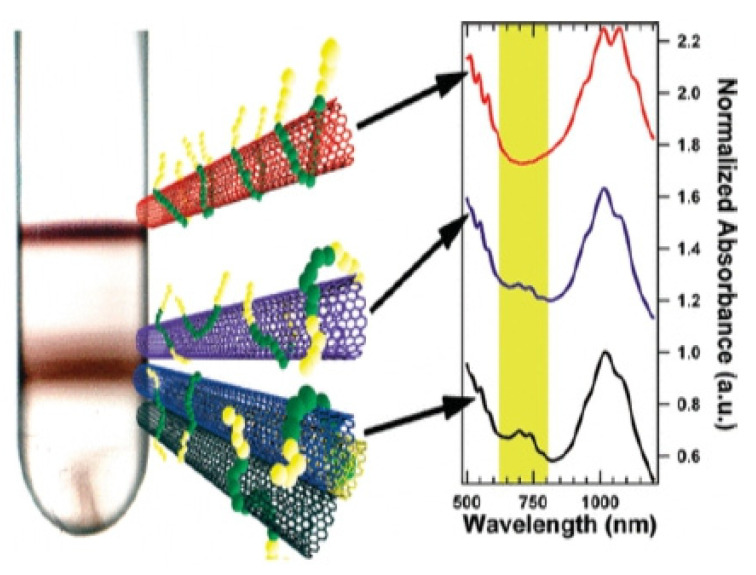
Centrifuge tubes after DGU separations using Pluronic F108 (top) and Pluronic F68 (middle) are shown in pictures. Optical absorbance spectra of the SWNTs that were extracted in the centrifuge tube at the locations shown by arrows [188]. The colors of CNTs represent their color in suspension.

The functionalized CNTs have been used in numerous applications. Here, we focus on some in which functionalization is the key to achieve a certain application. In particular, surfactant, used for functionalization, helped to sort SWCNTs by chirality and electronic types. First of all, the complete separation can be achieved if SWCNTs are present in the suspension as individual nanotubes. Secondly, the micelles created by the surfactant help to control the density and therefore, are useful in density gradient centrifugation (DGC), chromatography and two-phase separation. In [187], it was shown that the equal concentrations of surfactant, SDS and SC, results in distinct metal–semiconductor separation using DGC. Later work performed by Antaris et al. showed that metallic and semiconducting nanotubes can be sorted separately using X-shaped block copolymer Tetronic 1107 and the linear block copolymer Pluronic F68, respectively [188]. The non-ionic, biocompatible block copolymers can be used to isolate semiconducting and metallic SWNTs using density gradient ultracentrifugation. Pluronics with shorter hydrophobic chain lengths (Figure 8, top nanotubes) lead to a higher purity semiconducting SWNT layer formation in the centrifuge tube, while X-shaped Tetronic block copolymers (Figure 8, middle nanotubes) display an affinity for metallic SWNTs. This observation is proven by absorption spectroscopy measurement spectra consisting of unique absorption features associated with semiconducting and metallic SWNTs (Figure 8, right). The SWCNTs dispersed in a combination of surfactant such as SDS, DOC and SC separated them not only by the diameters, but also by chirality in the two-phase separation technique [189]. In addition, spontaneously aligned SWCNTs prepared by vacuum filtration have to be highly dispersed to prevent the formation of aligned bundle monodomains. In particular, SWCNTs functionalized by m-polyaminobenzene sulfonic acid was used and showed good dispersion in water and therefore, high order alignment was archived [190].

**Figure 9:**
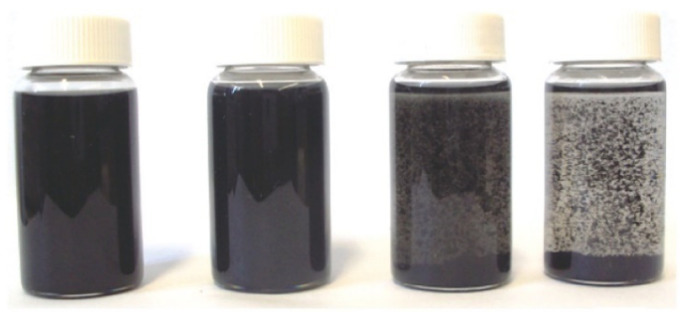
Photographs of various CNT dispersion in dilute epoxy solution (xylene-butanol (1:1) mixture) when the mixture was allowed to settle for 6 h. From left to right: CNT-ODA; CNT-OH; CNT-COOH and CNT-raw Copyright 2010, American Chemical Society. Reprinted with permission from Ref. [193]. Copyright 2004 American Chemical Society.

CNTs have further been utilized as a polymer matrix filler material. In the polymer matrix, the functionalized CNTs produce improved homogeneity and dispersion. It was demonstrated that interactions between CNTs and a polymer matrix are necessary for efficient CNT integration [191,192]. The colloidal behavior of the different functionalized CNTs, including carboxylated, octadecyl amide-functionalized and hydroxylated CNTs, was investigated in [193]. While −COOH and −OH are strongly polar, CNT-ODA is extremely stable in mixed polar−nonpolar solvents because of the amide groups (−CO-NH-) and nonpolar octadecyl groups (−C18H37). According to Figure 9 the CNT-COOH dispersion was extremely steady throughout the course of six hours. The dispersed amine-functionalized CNTs were analyzed by SEM after being mixed with the resin matrix. On the resin’s surface, there were some CNTs that weren’t functionalized, which shows how vital functional groups are for dispersion in epoxy resin [194]. Different sets of functionalization groups and polymers made using different processes were examined in the collection of papers published by Mallakpour et al. [195]. For instance, using a microwave-assisted technique, carboxylated MWCNT was covalently functionalized with 5-aminoisophthalic acid, increasing its solubility and dispersibility. In a different study, they revealed that S-valine amino acid was covalently attached to carboxylated MWCNTs via an amide bond and successfully dispersed in different aromatic polymers.

**Figure 10:**
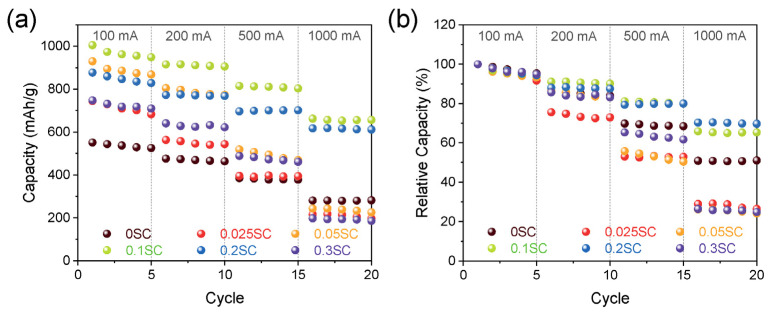
(**a**) Rate performance at a current density ranging from 100 to 1000 mA/g of SnO_2_@CNTF and (**b**) relative values of the capacity [196]. Colours showed different specific capacities.

In electrochemical energy storage technologies, such as lithium-ion batteries, supercapacitors, solar cells and fuel cells, functionalized CNTs have been thoroughly researched. Low-density CNTs demonstrate outstanding high-rate capabilities and cycle performance, according to tests on composite LiCoO_2_. For instance, SnO_2_-functionalized CNT derived from CNT functionalized with sulfonic acid was employed in lithium-ion battery anodes without the usage of a binder, conductive agent or current collector [196,197]. In this study, the SnO_2_@CNTF’s lithium storage capacity was determined using galvanostatic charge/discharge under the circumstances of a 100, 200, 500 and 1000 mA/g rate (Figure 10a,b). The SnO_2_ particles created in this experiment have superior rate capability and cycle stability compared to pure silicon particles [196].

As materials for electrodes, CNTs have been extensively studied. Various organic and inorganic compounds have been successfully coated on CNTs to enhance the electrode performance. When functionalizing CNT electrodes for the detection of glucose, urea and bisphenol A, noble metals like gold and silver nanoparticles have been utilized [198,199,200]. Numerous studies have been conducted on the enzyme sensors based on the electrodes of CNTs functionalized with nanomaterials including gold, platinum and graphene [201,202,203]. It was stated that an electrode for the detection of dopamine and uric acid has been developed using a combination of graphene foam, CNTs and gold nanoparticles [204]. pn junctions are produced for some applications by partially doping the CNT sheet [205]. While the p-type film is doped with benzylviologen, the n-type area is created by doping the as-grown material.

**Figure 11:**
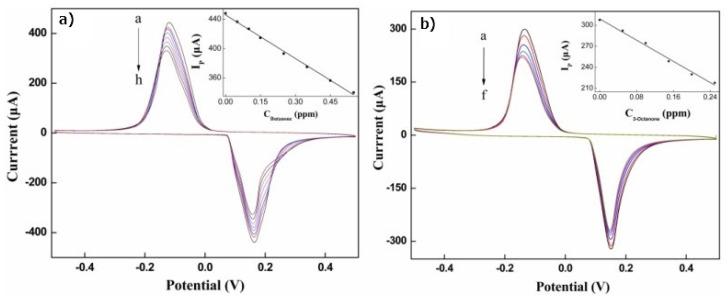
(**a**) The cyclic voltammograms of MWNTs/Au-Ag/GCE (butanone was varied from a to h: 0, 0.05, 0.1, 0.15, 0.25, 0.35, 0.45 and 0.55 ppm, respectively). (**b**) The cyclic voltammograms of MWNTs/Au-Ag/GCE under different concentrations of 3-octanone (varying from a to f: 0, 0.05, 0.1, 0.15, 0.2 and 0.25 ppm, respectively). The inset shows a line plot of the peak current (Ip) against the concentration of butanone and 3-octanone [206].

SWCNTs have been extensively explored for gas sensing applications because of their distinctive electrical characteristics. In order to preserve the increased contact with the detectable molecules caused by the enhanced surface interaction, it is crucial to solubilize and isolate CNTs for this purpose. Many research studies have functionalized the surface of SWNTS with different sensing materials in order to further improve the sensing performance, including the sensitivity and specificity (e.g., metallic nanoparticles, macromolecules, polymeric materials and bioreceptors, etc.). Different concentrations of butanone and 3-octanone were found using the electrochemical sensor in the N_2_ matrix [206]. As can be seen, a steady reduction in the anodic peak current (Ip) was detected with a negative shift in potential as the concentration of butanone increased (Figure 11a,b). Additionally, the MWNTs/Au-Ag/GCE electrochemical test method enables the detection of volatile biomarkers linked to stomach cancer cells. At a normal temperature, functionalized CNT exhibits a superior sensing performance to virgin samples and a strong sensitivity toward several chemicals, including benzene [207]. The detection of environmental air pollutants is one of the prominent directions where carbon nanotubes have been extensively applied [208].

Additionally, the functionality can lower the toxicity and improve the biocapability. Reduced blood circulation time, reticuloendothelial system uptake and other effects are caused by PEGylated CNTs [209]. It was also demonstrated that PEGylation did not impede SWCNT absorption into cells when the PEG chain’s molecular weight was less than 2000. The biological biocompatibility of PEG grafted to lipidophilic polymers is superior [210]. Oligothiophene-terminated PEG was used to effectively disperse CNTs in aqueous solution [210]. Particularly, the investigation focused on the important organs of mice injected intravenously with PEG-SWCNTs or non-PEG-SWCNTs. Following injection with oxidized SWCNTs, the lung tissue of control mice is shown in Figure 11a. There are concentrations of black, particulate particles that are probably not PEG-SWCNTs embedded in the lung parenchyma. In Figure 11b, black granular concretions can be seen in the lung tissue of mice that received injections of PEG-SWCNTs, however they seem to be smaller and fewer in number. Additionally, mononuclear reactive cells that are almost certainly macrophages surround these clumps [211].

The encapsulation of materials in CNTs has attracted interest. Metals and compounds have been placed into CNTs and can be manipulated using magnetic fields to target cells [212]. Furthermore, the endohedral functionalization can be used in targeted drug delivery and biomedical imaging [212,213]. SWCNTs filled with DNA and anticancer drugs such as hexamethylmelamine, irinotecan, indole and cisplatin were effectively used for targeted and efficient drug delivery. The release of drugs at the target can be triggered by electric stimulation, the pH of the medium or temperature. Magnetic resonance imaging used CNT encapsulated with magnetic atoms in a contrast agent such as gadolinium (III) salts, iron oxide or nitroxide radicals. Liquid chromatography mass spectrometry analysis was used to determine the composition of the phospholipids that were adsorbed on the SWCNTs. Phosphatidylcholines (PC) and phosphatidylglycerols, the two most prevalent forms of surfactant phospholipids, were discovered to be preferentially adsorbed by SWCNTs [214].

High water transport rates, supported by functionalized carbon nanotube pores, closely resemble the structure of aquaporin channels, making them excellent candidates for the creation of artificial water channels and high-performance membranes [215,216,217]. Functionalized CNTs were also proposed for the adsorption and removal of contaminants. These functional groups affect the adsorption capability of CNTs by making CNTs more hydrophilic for efficient adsorption. In particular, CNT membrane—filters were investigated due to the known high mechanical robustness and high porosity. Recently, various chemical modification methods were used for CNTs (fluorination, cycloaddition, reaction with diazonium salt, oxidation and radical polymerization) to enhance the CNT’s solubility and reactivity to water remediation [218]. A variety of MWCNT-modified electrospun membranes were developed to immobilize enzymes. CNTs have also received significant attention in fuel cell treatment [197].

## 5. Conclusions

The chemistry of carbon nanotubes represents a serious topic in science and technology. Modification of the outer surface is performed by covalent attachment of functional groups. Here, it is considered with transmission electron microscopy, scanning tunnelling microscopy, Raman spectroscopy and X-ray photoelectron spectroscopy. Modification of the outer surface of carbon nanotubes by the wrapping and adsorption of molecules is an important technique. The substitution of atoms into carbon nanotubes via other atoms is a way to tailor the physical properties of nanotubes. The filling of carbon nanotubes opens up a way to study the chemistry and physics of carbon nanotubes. The demonstrated applications of functionalized CNTs are presented.

## Data Availability

Not applicable.

## Abbreviations

The following abbreviations are used in this manuscript:

CNT	Carbon nanotube
CTAB	Cetyltrimethyl ammonium bromide
CVD	Chemical vapor deposition
DGC	Density gradient centrifugation
DGU	Density gradient ultracentrifugation
DNA	Deoxyribonucleic acid
DOC	Sodium deoxycholate
EELS	Electron energy loss spectroscopy
F-SWCNT	Fluorinated single-walled carbon nanotube
FTIR	Fourier-transformed infrared spectra
GCE	Glassy carbon electrode
MWCNT	Multi-walled carbon nanotube
NP	Nanoparticles
PNA	Peptide nucleic acid
SC	Sodium cholate
SDBS	Sodium dodecyl benzene sulfonate
SDS	Sodium dodecyl sulphate
siRNA	Small interfering ribonucleic acid
STEM	Scanning transmission electron microscopy
STM	Scanning tunnelling microscopy
SWCNT	Single-walled carbon nanotube
TEM	Transmission electron microscopy
TX	Triton X
UV	Ultraviolet
XPS	X-ray photoelectron spectra
